# Single-cell analysis unveils cell subtypes of acral melanoma cells at the early and late differentiation stages

**DOI:** 10.7150/jca.102045

**Published:** 2025-01-01

**Authors:** Mengyuan Hou, Zhijie Zhao, Shuxiao Li, Ziwei Zhang, Xin Li, Yichi Zhang, Wenyi Huang, Li Li, Wenjing Xi, Feiteng Liang, Li Lin, Yan Zhang, Gang Chai

**Affiliations:** 1 Department of Plastic and Reconstructive Surgery, Shanghai 9th People's Hospital, School of Medicine, Shanghai Jiao Tong University, 639 Zhi Zao Ju Rd, Shanghai, 200011, China.; 2 Department of Burns and Plastic Reconstructive Surgery, Affiliated Hospital of Youjiang Medical University for Nationalities, Baise, 533000, Guangxi Province, China.; 3 Life Science and Clinical Medicine Research Center, Affiliated Hospital of Youjiang Medical University for Nationalities, Baise, 533000, Guangxi Province, China.

**Keywords:** melanoma, acral melanoma, scRNA-seq, PCLAF+ melanoma cells

## Abstract

Background: Melanoma, a malignant neoplasm originating from melanocytes, is a form of skin cancer with rapidly increasing global incidence, often exacerbated by UV radiation[1]. Particularly, acral melanoma, characterized by its swift metastasis and poor prognosis, underscores the significance of further research into its heterogeneity. Single-cell sequencing has been widely utilized in the study of tumor heterogeneity; however, research related to melanoma remains to be further refined.

Materials and Methods: We employed single-cell RNA sequencing (scRNA-seq) transcriptomic analysis to delve into the melanoma cells from six samples of melanoma patients. This approach enabled the identification of critical melanoma cell subpopulations and their roles in melanoma progression. Subsequently, we examined the interactions among these subpopulations and analyzed their interactions with other cell types.

Results: Our analysis identified C3 ID2+ melanoma cells as an early-stage subpopulation and C4 PCLAF+ cells as a late-stage subpopulation in melanoma evolution. Through our analysis, we identified C4 PCLAF+ Melanoma cells as a significant subpopulation in acral melanoma (AM), playing a pivotal role in the differentiation and development of AM. Further analysis of transcription factors, enriched pathways, cell stemness, and cell trajectories highlighted the significant role of C4 PCLAF+ melanoma cells in acral melanoma (AM) proliferation.

Conclusion: This study identifies new factors influencing melanoma progression, providing a foundation for subsequent research.

## Introduction

Acral melanoma (AM) is a rare subtype that primarily affects the glabrous skin of the palms, soles, and nail unit, and it is associated with a significantly poorer prognosis compared to other forms of cutaneous melanoma^2^. Despite this, its genetic underpinnings, encompassing unique genomic drivers and causal factors, have received limited exploration^3^. Current evidence suggests that acral melanomas follow a tumorigenesis pathway independent of UV radiation, characterized by fewer single nucleotide mutations than those found in other cutaneous melanomas. Furthermore, comprehension of alternative therapeutic targets for acral melanoma remains incomplete^4^, ^5^.

Many studies have employed single-cell sequencing in the investigation of tumor heterogeneity^6^, ^7^. Simultaneously, the melanoma tumor microenvironment is composed of various cell types, including tumor cells, immune cells, fibroblasts, and endothelial cells. Single-cell sequencing offers an unprecedented opportunity to investigate melanoma tumor heterogeneity. Zhang and colleagues reported that PD1 and TIM-3 exhibit elevated expression levels in exhausted CD8+ T cells specific to acral melanoma^8^. Additionally, Smalley *et al.* identified a rare dendritic cell population (DC3) associated with improved overall survival and a positive modulation of the immune microenvironment^9^. Huuhtanen *et al.* observed heightened baseline TCR clonality in patients responding to combination therapy involving anti-LAG-3 and anti-PD-1, with their expanding CD8+ T cell clones acquiring a more cytotoxic and NK-like phenotype^10^. Nonetheless, a subset of melanoma patients fails to derive benefits from immunotherapy or molecular-targeted treatment modalities, leaving their disease management challenging. Despite ongoing advancements in treatment options, the prognosis for melanoma patients remains unfavorable^11^. It is worth noting that acral melanoma is more likely to metastasize and has a worse response to treatment^4^. Cancer progression is governed by complex intracellular signaling networks, where multiple pathways intersect to regulate tumor cell phenotypes^12^-^14^. Therefore, elucidating the underlying mechanisms driving melanoma progression is essential for refining therapeutic approaches and ultimately improving patient outcomes.

This study utilized single-cell RNA sequencing (scRNA-seq) on melanoma samples to unravel the tumor heterogeneity of melanoma. Further investigation into melanoma cells could yield fresh perspectives on melanoma therapy, potentially improving the prognosis of melanoma cells. The paper examined the functional roles of melanoma cell subpopulations throughout melanoma progression, offering new insights and perspectives for future research and clinical applications in the field of melanoma^15^-^18^.

## Materials and Methods

### Data source

The study began by examining publicly available single-cell RNA sequencing data from the Gene Expression Omnibus (GEO) for a thorough analysis. To address melanoma's inherent heterogeneity, the c dataset, encompassing various subtypes and diverse cell types, was selected to thoroughly understand their biological characteristics. Additionally, we obtained the clinical information related to this dataset, which aids us in the further analysis and processing of the data^19^.

### Analysis of single-cell RNA sequencing data

The raw scRNA sequencing data were processed using the "Seurat" package (version 4.3.0.1) and analyzed with the "DoubletFinder" package (version 2.0.3) to identify potential doublets^20^. Cells were excluded if they had fewer than 300 or more than 6000 genes; fewer than 500 total molecules (nCount_RNA); more than 100,000 total molecules; more than 20% of their genes; or with over 5% mitochondrial gene expression^21^, ^22^.

For each sample, the gene expression was represented as the fraction of the gene and multiplied by 10,000, which were converted into natural logarithm and normalized after adding 1 to avoid taking the log of 0. The normalized expression matrix was used to find the top 2000 highly variable genes (HVGs). Then, we scaled them before running a principal component analysis (PCA) on these genes. Using the R Harmony package (version 1.0), batch effects were eliminated based on the top 30 PCA components^23^. Based on harmony-corrected data, k-nearest neighbors (KNN) were calculated, and a shared nearest neighbor (SNN) graph was constructed. The modular function was then modified based on the clustering algorithm to accomplish cluster recognition. The identified clusters were presented on the 2D map made with the uniform manifold approximation and projection (UMAP) for dimension reduction method^24^.

### Clustering and cell type identification

Using the “FindAllMarkers” function, we identified the marker genes for each cluster based on the following parameters: logfc.threshold = 0.25, min.pct = 0.25, and min.diff.pct = 0.25. The DotPlot and FeaturePlot functions in Seurat were used to visualize the expression patterns of marker genes across clusters. Based on the DEGs and well-known cellular markers mentioned in the prior research^19^, ^25^, the cell groups were annotated. Additionally, to further investigate melanoma cell heterogeneity, the melanoma cells were re-clustering. Each subgroup of melanoma cells was then labeled based on its distinctive genes.

### Enrichment analysis of GO-BP and GSEA

The “ClusterProfiler” software package (version 0.1.1) was used to conduct the GO analysis of differentially expressed genes (DEGs) in each subpopulation. The GO Biological Processes (GO-BP) database hosted at http://www.geneontology.org was utilized for this purpose^25^.

Gene set enrichment analysis (GSEA) employed the MSIGDB database from the GSEA website to explore gene functions in greater depth. The gene expression matrices of melanoma patients were analyzed separately using the limma R package (version 3.54.2)^26^. P-values were adjusted for false discovery rate (FDR), with a significance threshold set at p < 0.05.

### Cell stemness analysis

The AUCell package was used to score pathways in each cell based on gene set enrichment analysis (GSEA)^27^.

### Trajectory analysis

Two pseudotime analysis tools, Monocle (version 2.22.0)^28^ and Slingshot (version 2.6.0)^29^, were utilized to study melanoma progression.

The pseudotime trajectories of melanoma cells were analyzed using the Monocle package (version 2.22.0). Using pseudotemporal profiling of scRNA-seq data, Monocle aims to identify cellular alterations that occur during differentiation of melanoma cells. After inserting the scale of raw UMI counts into the “newCellDataSet” function with its clustering information, it was transformed into a reduced dimensional space using the discriminative dimensionality reduction with trees (DDRTree) technique, a more current manifold learning method. According to pseudotime, melanoma cells were then arranged. The plot pseudotime heatmap was used to identify and display the genes whose expression varied simultaneously with pseudotime^30^.

The utilization of the “Slingshot” package (version 2.6.0) allows for the integration of robust methodologies suitable for dealing with noisy single-cell data, along with the capacity to detect and delineate multiple trajectories. This package effectively combines techniques that provide stability and reliability in the analysis of single-cell datasets characterized by inherent variability and uncertainty. Through its capabilities, “Slingshot” enables the identification and characterization of distinct developmental or differentiation trajectories within the cellular landscape^31^, ^32^. The “getlineage” function was used to infer cell lineages and observe trajectories in different subgroups, and the “getCurves” function was used to estimate cell expression levels.

### Single-cell regulatory network inference and clustering for Python

We used the pySCENIC R (version 0.10.0) package to analyze transcription factors differentially expressed in various subpopulations identified through scRNA-seq^32^. The steps of pySCENIC can be divided into three stages. First, co-expression modules are inferred using the regression method for each target (GRNBoost2). Next, indirect targets are removed from these modules using cis-regulatory motif discovery (cisTarget). Finally, the activity of these regulators is quantified by the enrichment scores of the regulon target genes (AUCell). Nonlinear projection methods can visually group cells based on the activity patterns of these regulons^33^.

### scMetabolism analysis

scMetabolism (v0.2.1) was used for visualisation and quantifying the metabolic diversity of single cells in each cluster. ScMetabolism used vision algorithm to score each cluster, and finally obtained the activity score of clusters in each metabolic pathway based on the conventional single-cell matrix file^34^.

### Examining interactions between cells

Exploring the network of interactions between different subgroups of epithelial cells using the "CellChat" package (version 1.6.1), ligand-receptor pairs between cells derived from melanoma patients were explored^35^. CellChat contains a comprehensive signaling molecule interaction database that takes into account the known structural composition of ligand-receptor interactions infers cell-state specific signaling communications within a given scRNA-seq data using mass action models^1^. To predict cell-cell interactions among the different cell types, a significance threshold of 0.05 (P-value) was employed.

## Results

### scRNA sequencing revealed the main cell types in the progress of melanoma

In order to obtain the main cell types in melanoma, we collected 5 acral and 3 cutaneous tumor specimens from 6 melanoma patients to obtain scRNA-seq (**Figure S1A**). There are seven primary and one lymph node metastatic tumor samples. We collected the pre- and post-treatment samples from one patient who received immunotherapy. After initial quality control and batch effect removal, a total of 83741 cells were retained. We clustered these 83741 cells by dimensionality reduction, and the analysis revealed 36 unique tissue states (**Figure S1B-D**). Based on the typical tissue type-specific markers defined in the literature, we divided the cell clusters into six main cell types: Melanoma cells, T_NK cells, Fibroblasts, ECs, Myeloid cells and B_Plasma cells.

### Visualization of melanoma cell subgroups

**Figure 1:**
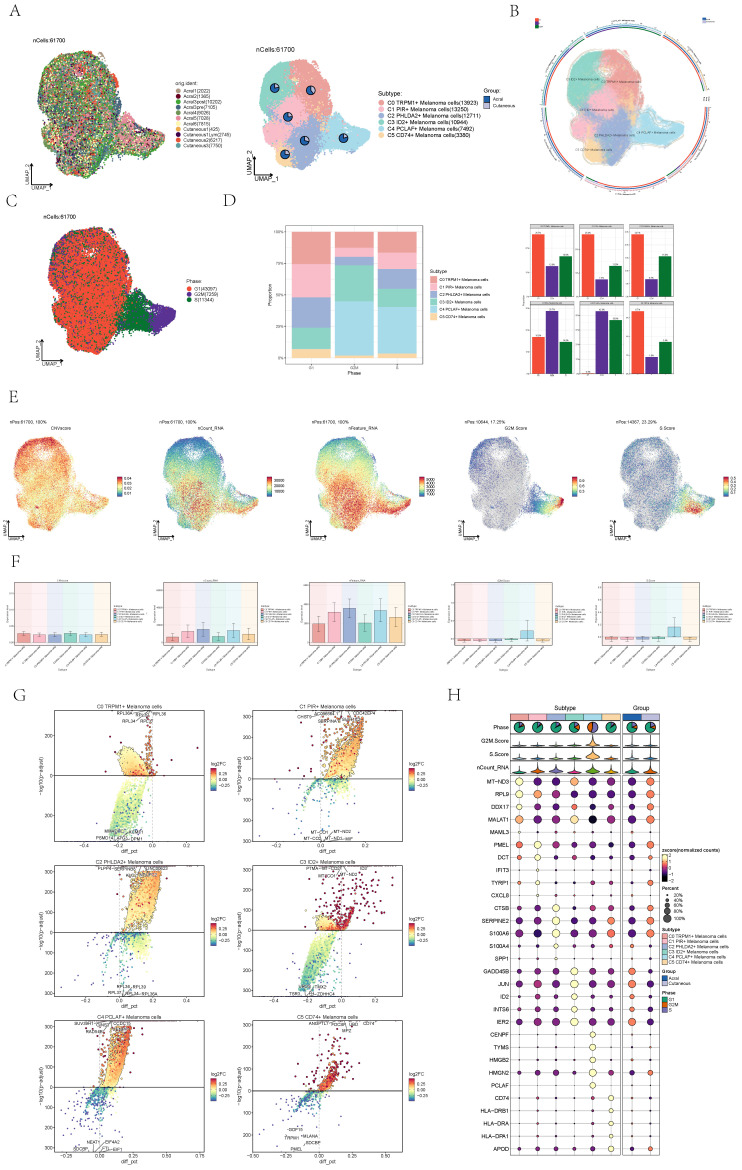
** Visualization of melanoma cell subpopulations. (A)** UMAP diagram demonstrated the patient origin of melanoma cells in melanoma patients and the number of cells in each group (left); UMAP diagram demonstrated the distribution of the Acral group and the Cutaneous group in the 6 cell subpopulations (right).** (B)** UMAP diagram demonstrated the distribution of different groups and phases in the 6 cell subpopulations.** (C)** UMAP diagram demonstrated the 3 phases of melanoma cells in melanoma patients and the number of cells in each group.** (D)** Bar graph showed the percentage of each cell subpopulation at different tumor stages (left); bar graph showed, in each cell subpopulation, the percentage of cells with different cell cycles (right). **(E)** UMAP plots visualized the relevant features of 6 cell subpopulations: CNVscore, nCount_RNA, nFeature_RNA, S.score, G2M.score. **(F)** Bar graph showed the relevant features of 6 cell subpopulations: CNVscore, nCount_RNA, nFeature_RNA, S.score, G2M.score.** (G)** Volcano plots demonstrated the expression of differential genes in 6 cellular subpopulations.** (H)** Bubble graph showed differential expression of Top10 maker genes in 6 cell subpopulations of melanoma cells. The color of the bubbles was based on the normalized data and the size indicated the percentage of genes expressed in the subpopulation.

Next, we distinguished melanoma cells and made further sub-clustering, resulting in six cell subgroups and marking their cell numbers: C0 TRPM1+ Melanoma cells (13923), C1 PIR+ Melanoma cells (13250), C2 PHLDA2+ Melanoma cells (12711), C3 ID2+ Melanoma cells (10944), C4 PCLAF+ Melanoma cells (7492), C5 CD74+ Melanoma cells (3380) (**Figure 1A**), and demonstrated the proportion of cells in 6 cell subtypes (**Figure 1B**). Compared to other subgroups, melanoma cells in C4 exhibited the highest proportion of acral melanoma group within this subgroup. Therefore, it is needed to further explore features of C4 from other aspects such as the gene expression and cellular communication by single-cell sequencing.

We presented the distribution of cells at different phases of the cell cycle across various subsypes, wherein the cells within the C4 subtype were predominantly in the G2M and S phase (**Figure 1C**). The proportion of cells within the C4 subtype was also higher in the S phase and G2 phase compared to other subgroups (**Figure 1D**). The higher proportion of cells in the G2M and S phase within C4 suggested a greater proliferative capacity. Several related features (CNV score, nCount_RNA,nFeature_RNA, S. score and G2M. score) of six cell subgroups were visualized by UMAP diagrams (**Figure 1E**) and bar plots (**Figure 1F**). Consistently, the S score and G2M score of the C4 subsype were significantly higher than those of other subtypes. As previously reported, AM is characterized by a propensity for rapid dissemination and high metastatic potential^36^. Given the strong proliferative capacity of the C4 subgroup, it may play a significant role in the rapid dissemination and high metastatic potential characteristic of AM.

The differential genes of six cell subtypes were displayed by volcano diagrams (**Figure 1G**), and the marker genes (top10) in each cell subtype were displayed by bubble diagram (**Figure 1H**).

### Visualization of enrichment analysis of melanoma cells

**Figure 2:**
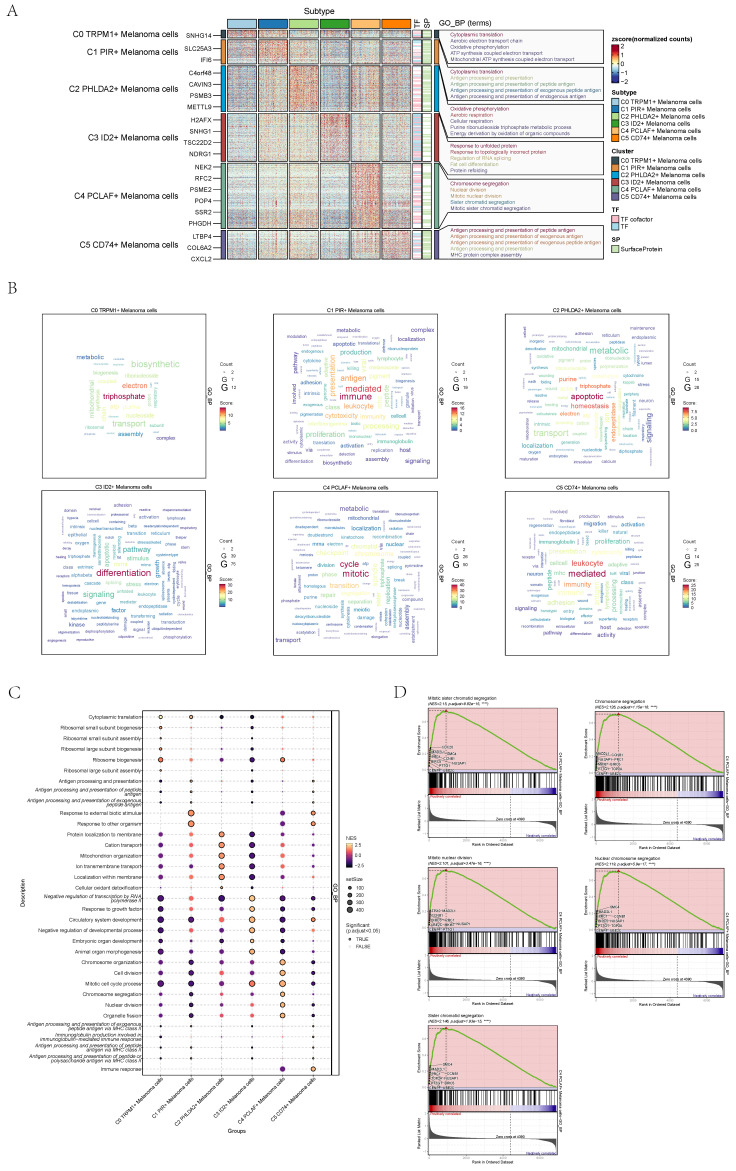
** Visualization of GO analysis of melanoma cells. (A)** GO-BP enrichment analysis demonstrated the biological processes associated with the 6 cell subpopulations. **(B)** Word cloud diagrams showed gene pathway enrichment in 6 cell subpopulations. The size of the letters indicated the number of enriched pathways, and the color indicated the high or low score of enriched pathways in different cell subpopulations. **(C)** Bubble graph showed GO-BP enrichment analysis of 6 cell subpopulations. The color of the bubbles was based on the normalized data and the size indicated the percentage of genes expressed in the subpopulation.** (D)** Enrichment score values on different pathways in C4 cluster were displayed by GSEA scoring of GO-BP enrichment entries for differential genes.

The results of enrichment analysis of GO-BP, the differential genes of six cell subgroups, were displayed by thermogram (**Figure 2A**). The enrichment pathways were displayed by word cloud diagrams (**Figure 2B**) and dotplots (**Figure 2C**).

C0 TRPM1+ Melanoma cells were related to biological processes such as Cytoplasmic translation, Aerobic electron transport chain, Oxidative phosphorylation, etc. C1 PIR+ Melanoma cells were related to biological processes such as Cytoplasmic translation, Antigen processing and presentation, Antigen processing and presentation of peptide antigen, etc. C2 PHLDA2+ Melanoma cells were related to biological processes such as Oxidative phosphorylation, Aerobic respiration, Cellular respiration, etc. C3 ID2+ Melanoma cells were related to biological processes, such as Response to unfolded protein, Response to topologically incorrect protein, Regulation of RNA splicing, etc. C4 PCLAF+ Melanoma cells were related to biological processes such as Mitotic sister chromatid segregation, Mitotic nuclear division, Sister chromatid segregation, nuclear chromosome segregation AND Chromosome segregation, etc. (**Figure 2D**). C5 CD74+ Melanoma cells were related to biological processes such as Antigen processing and presentation of peptide antigen, Antigen processing and presentation of exogenous antigen, Antigen processing and presentation of exogenous peptide antigen, etc. The results of enrichment analysis further corroborated the potent proliferative capacity of C4.

### Visualization of cell stemness of melanoma cells

**Figure 3:**
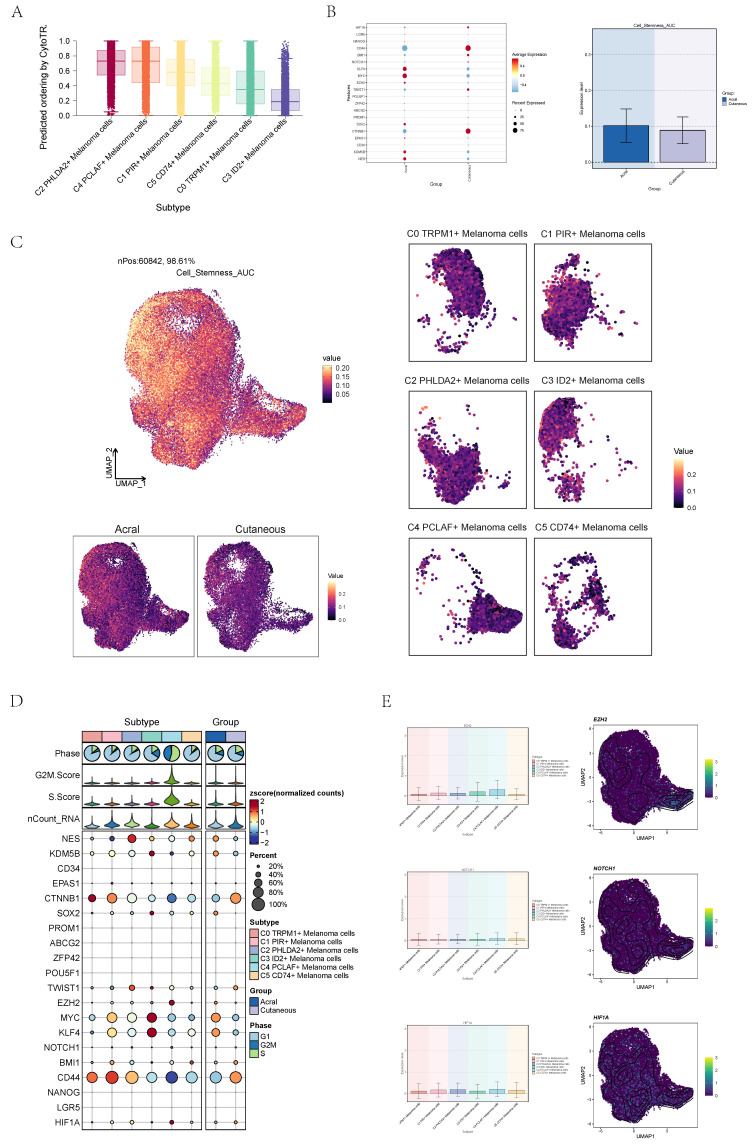
** Visualization of the cell stemness of each subpopulation of cells. (A)** Boxplots showing CytoTRACE values for different subtype of melanoma cells. Statistical significance was assessed by a two-sided Wilcoxon signed-rank test. **(B)** Bubble graph and bar graph showed Cell stemness-related AUC value of cell stemness score of melanoma cells in acral melanoma and cutaneous melanoma. **(C)** UMAP diagram showed Cell stemness-related AUC calculated by the AUCell function in all melanoma cells (top left) and different groups (bottom left) and subtypes (right). The color of was based on value.** (D)** Bubble graph showed differential expression of cell stemness-related genes in 6 cell subpopulations of melanoma cells. The color of the bubbles was based on the normalized data and the size indicated the percentage of genes expressed in the subpopulation.** (E)** UMAP diagram and bar graph showed expression of cell stemness-related genes in 6 cell subpopulations of melanoma cells.

Cancer stemness of each subtype was evaluated by CytoTRACE and the results indicate that C4 cells possessed high cell stemness and The C3 cells exhibited the lowest cell stemness (**Figure 3A**). Subsequently, the cell stemness of AM and CM cells was compared, revealing a higher degree of stemness in AM cells (**Figure 3B**). AUC of cell stemness in all melanoma cells, different cell cycle stages, acral and cutaneous melanoma and six cell subgroups by UMAP diagrams (**Figure 3C**). The expression signatures of genes related to cell stemness in each cell subgroup were displayed by bubble diagram (**Figure 3D**). The expression level of several genes associated with cell stemness, including EZH2, NOTCH1 and HIF1A, were depicted in the graph UMAP plots and bar plots (**Figure 3E**). Compared to other subgroups, the expression levels of these genes were relatively higher in C4. From the various analyses conducted above, it was evident that C4 exhibited higher cell stemness, indicating that it may play a significant role in the growth and metastasis of AM.

### Visualization of pseudo-sequential analysis of melanoma cells by monocle

**Figure 4:**
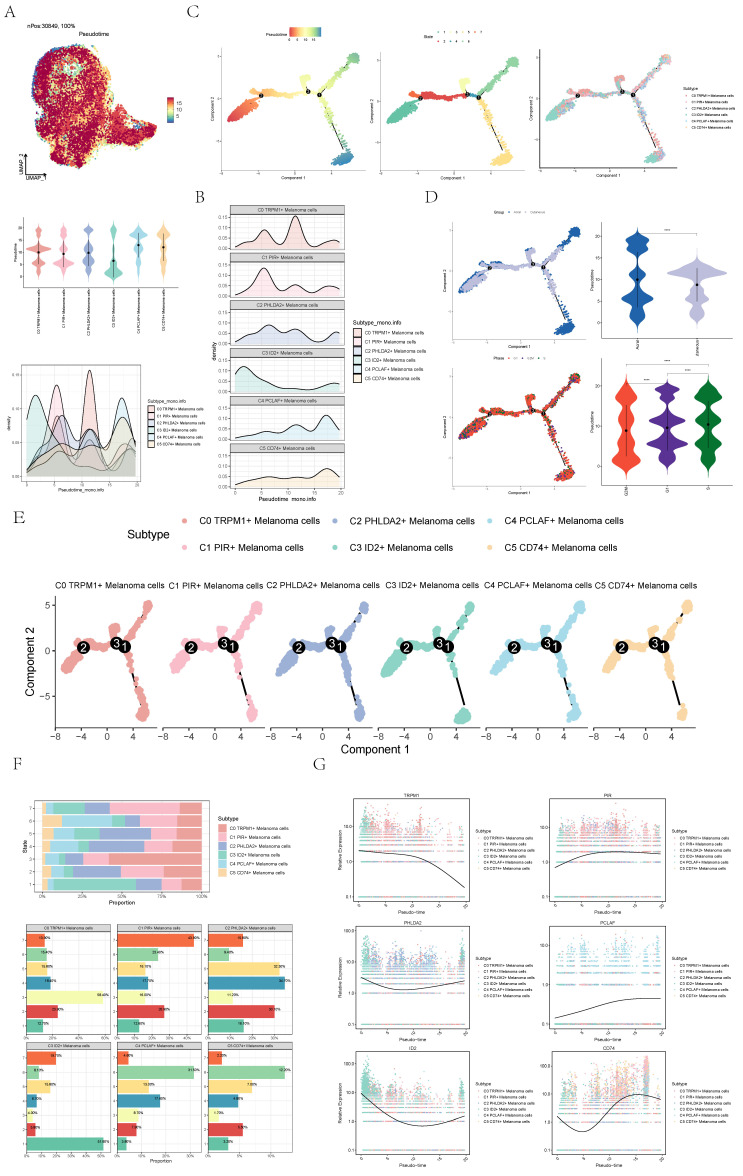
** Visualization of pseudotime-series analysis of melanoma cells. (A)** UMAP plot(top) and violin plot(bottom) showed the pseudotime distribution of melanoma cell subpopulations.** (B)** Ridge plot showed pseudotime distribution of melanoma cell subpopulations. **(C)** The derivation process of melanoma cells. Left: the figure showed the pseudotime trajectory of melanoma cells; middle: the pseudotime trajectory graph showed the distribution of STATE; right: the pseudotime trajectory graph showed the distribution of melanoma cell subpopulations. **(D)** The pseudotime trajectory graph(left) and violin plot(right) showed the distribution of melanoma cells in different groups (top) and phases (bottom). **(E)** Split-plane diagrams of melanoma cell pseudotime sequence trajectories showed the distribution of different cell subpopulations on the pseudotime sequence, respectively. **(F)** Bar graph showed the occupancy of different states (state1-state7) in 6 melanoma cell subpopulations.** (G)** Scatter plots showed the changes of named genes of 6 cell subpopulations of melanoma cells with the pseudotime sequence.

Pseudo-sequential analysis of cancer development process was carried out to explore the differentiation process of melanoma cells. The distribution of melanoma cells was shown in the pseudotime-series trajectory, and the distribution of cell subgroups in pseudotime-series was shown by using UMAP diagram (**Figure 4A**, top), violin diagram (**Figure 4A**, middle) and ridge diagram (**Figure 4B**) respectively. It could be seen that six cell subgroups were continuously differentiated in pseudotime-series and C4 predominantly occupied the terminal stage while C3 primarily occupied the early stages.

In order to study the progression of melanoma cells, the pseudotime sequence trajectory of six cell subgroups was further analyzed. Seven kinds of states were identified. Starting from state1 at the left of the trajectory, two trajectories are divided upward, one is state7 upward, the other is continuously divided upward to the second branch point of state2, and the second branch point was divided into two branches, one is state3 upward, and the other is state4 downward to the right, which divided upward to the next branch point. This point was divided into two branches, one is state6 upward, and the other is state5 downward (**Figure 4C**). The acral melanoma cells mostly at the early and late state of pseudotime series, while the cutaneous cells mostly at the middle state of pseudotime series. Melanoma cell in cell cycle stages basically run through the pseudotime series (**Figure 4D**).

The sectional views of each subgroup showed that the C4 subgroup was located towards the end of pseudotime series (**Figure 4E**). In state1, the proportion of C3 ID2+ Melanoma cells subgroup was the highest, while in state2, state4 and state5, the proportion of C2 PHLDA2+ Melanoma cells subgroup was the highest, and in state3, the proportion of C0 TRPM1+ Melanoma cells subgroup was the highest, and in state6, the proportion of C4 PCLAF+ Melanoma cells subgroup was the highest, and C1 PIR+ Melanoma cells subgroup only existed in state7. In C4 subgroups, the proportion of state6 was the highest (**Figure 4F**). The named genes of six cell subgroups were selected and their changes with pseudotime series were shown by scatter plots. It could be seen that gene PCLAF+, representing the C4 subgroup, was mostly at the end of pseudotime series. However, gene ID2+, representing the C3 subgroup, was mostly in the initial state of pseudotime series (**Figure 4G**). The sectional views of each subgroup also confirmed this conclusion.

### Slingshot analysis of pseudotime sequence trajectory of melanoma cell subgroups

**Figure 5:**
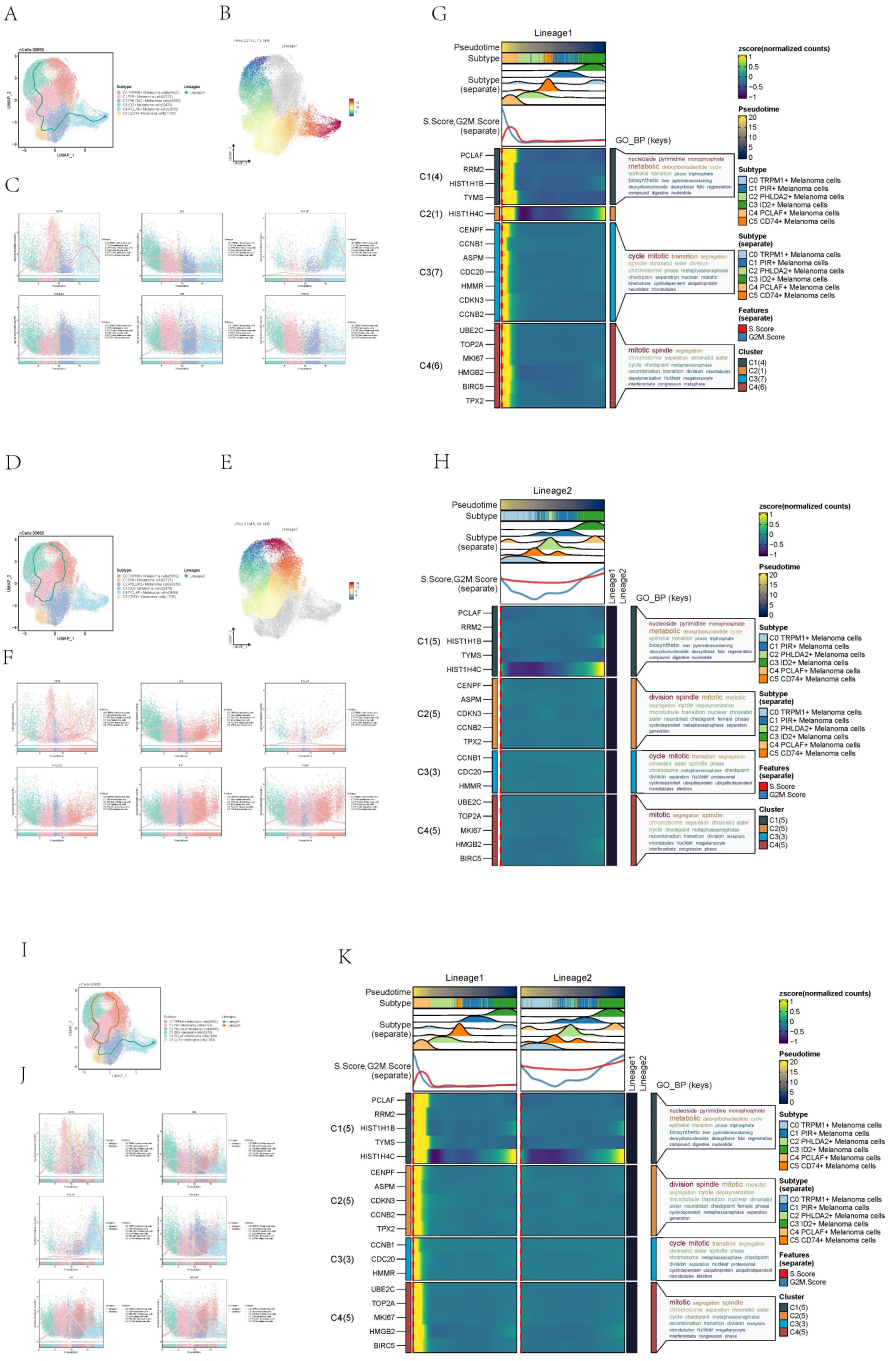
** Slingshot analysis of melanoma cell subpopulations on pseudotime trajectories. (A)** UMAP plot showed the distribution of Lineage1 trajectories of melanoma cells fitted by the pseudotime order in all melanoma cells. **(B)** UMAP plot demonstrated the change of Lineage1 with the fitted pseudotime order. **(C)** Scatter plots demonstrated the trajectories of named genes of 6 cell subpopulations of melanoma cells changing on Lineage1 obtained after slingshot visualization. **(D)** UMAP plot showed the distribution of Lineage2 trajectories of melanoma cells fitted by the pseudotime order in all melanoma cells. **(E)** UMAP plot demonstrated the change of Lineage2 with the fitted pseudotime order. **(F)** Scatter plots demonstrated the trajectories of named genes of 6 cell subpopulations of melanoma cells changing on Lineage1 obtained after slingshot visualization. **(G)** GO-BP enrichment analysis demonstrated the biological processes corresponding to Lineage1 pseudotime trajectories of melanoma cell subpopulations.** (H)** GO-BP enrichment analysis demonstrated the biological processes corresponding to Lineage2 pseudotime trajectories of melanoma cell subpopulations.** (I)** UMAP plot showed the distribution of two differentiation trajectories of melanoma cells fitted by the pseudotime order in all melanoma cells. **(J)** Scatter plots demonstrated the trajectories of named genes of 6 cell subpopulations of melanoma cells changing on Lineage1 and Lineage2 obtained after slingshot visualization. **(K)** GO-BP enrichment analysis demonstrated the biological processes corresponding to Lineage1 and Lineage2 pseudotime trajectories of melanoma cell subpopulations.

In order to infer the continuous branching pedigree structure in melanoma cell data, the pseudotime series trajectories of six cell subgroups were analyzed by using slingshot, and two lineages were obtained: lineage1 and lineage2. Next, the relationship between two lineages and pseudotime-series trajectory and pseudotime-series differentiation trajectory was displayed respectively, and the distribution status of different subpopulations on lineage1 and lineage2 and the differentiation curves with the pseudotime series were shown in scatter plots (**Figure 5A-F**). Clearly, C4 subgroup existed at the end of lineage1 while C3 subgroup existed at the end of lineage1 and lineage2.

The two pseudotime-series trajectories were visualized by GO-BP enrichment analysis. Then, it was found that C4 in lineage1 was related to biological processes such as mitotic spindle C1 was related to biological processes such as nucleoside pyrimidine monophosphate, and C3 was related to biological processes such as cycle mitoti (**Figure 5G**). In lineage2, C4 was related to biological processes such as mitotic, C1 was related to biological processes such as nucleoside pyrimidine, C2 was related to biological processes such as division spindle, and C3 was related to biological processes such as cycle mitotic (**Figure 5H**).

Finally, lineage1 and lineage2 were jointly displayed (**Figure 5I-K**). Pseudotime series analysis showed that C3 ID2+ Melanoma cells may be the initial stage of tumor cell proliferation while C4 PCLAF+ Melanoma cells may be the terminal stage of tumor cell proliferation, and Some C3 cells gradually differentiated into C4 during the progression of melanoma.

### Analysis of transcription factors in cell subgroups

**Figure 6:**
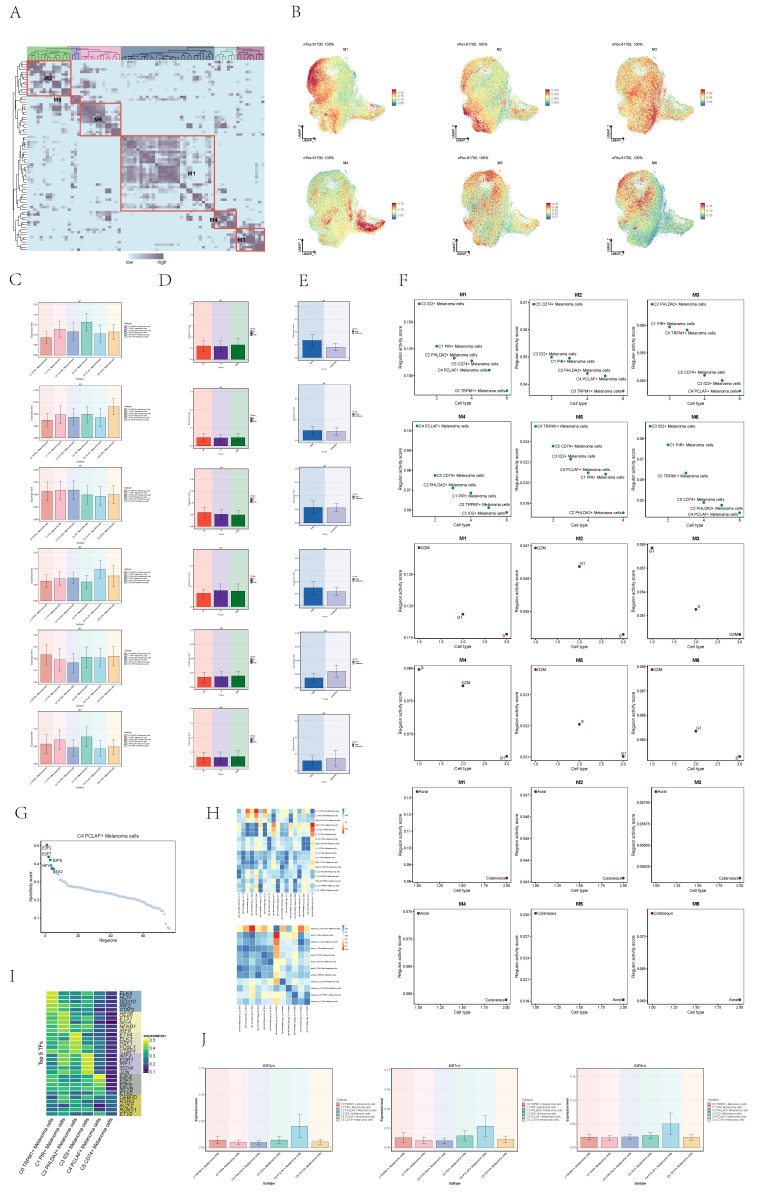
** Visualization of expression level of transcription factors of melanoma cells. (A)** Heatmap showed the relative activity of transcription factors in each of the 6 melanoma cell subgroups. Single-cell regulatory network inference and clustering (pySCENIC) for Python analysis was applied to obtained six models.** (B)** UMAP diagrams showed the expression level of each transcription factor modules in melanoma cells.** (C)** Bar graphs showed expression level of each transcription factor modules in 6 melanoma cell subtypes.** (D)** Bar graphs showed expression level of each transcription factor modules in melanoma cells in different phases. **(E)** Bar graphs showed expression level of each transcription factor modules in melanoma cells in different groups. **(F)** Scatter plot showed regulon activity score of each transcription factor modules in different subtypes(top), groups(middle) and phases(bottom) melanoma cells.** (G)** Dotplot showed the specificity score of transcription factor in C4 PCLAF+ Melanoma cells subgroup.** (H)** Heatmaps showed the pairwise Pearson correlation coefficients of transcription factor expression level among different groups of cells. **(I)** Heatmap showed the The top five transcription factors by expression level in six cells subgroups.** (J)** Bar graphs showed expression level of 3 transcription factors in 6 melanoma cell subtypes.

To further analyze the heterogeneity of melanoma cell subgroups, we used single-cell regulatory network inference to infer the transcription factor (TF) of melanoma cells, conducted clustering of TFs and obtained six modules (**Figure 6A**). The regulon activity score of each module in melanoma cells is displayed collectively in the UMAP plots (**Figure 6B**).

Compared to other cell subpopulations, the expression levels of transcription factors in Module 4 (M4) are highest in the C4 subpopulation (**Figure 6C**). Simultaneously, the expression levels of M4 are higher in S phase and G2M phase compared to G1 phase (**Figure 6D**), and in acral group compared to cutaneous group (**Figure 6E**). The regulon activity score of M4 is consistent with the expression level results (**Figure 6F**). Acral_C4 PCLAF+ Melanoma exhibited a higher degree of similarity to all acral melanoma cell groups. Considering acral melanoma samples have significantly severe immunosuppressive state compared with cutaneous melanoma^36^, acral melanoma cells may collectively exhibit high expression of certain transcription factors, leading to the manifestation of this characteristic.

Next, we further analyzed the transcription factors that played crucial roles in the C4 melanoma subgroup. The expression levels of E2F2, E2F7, and E2F8 in the C4 melanoma cell subgroup were significantly higher compared to other subgroups (**Figure 6G-J**). These transcription factors are associated with the proliferation and metastasis of various types of human cancers, particularly skin cancer^37^-^41^.

### Metabolic analysis of cell subgroups

**Figure 7:**
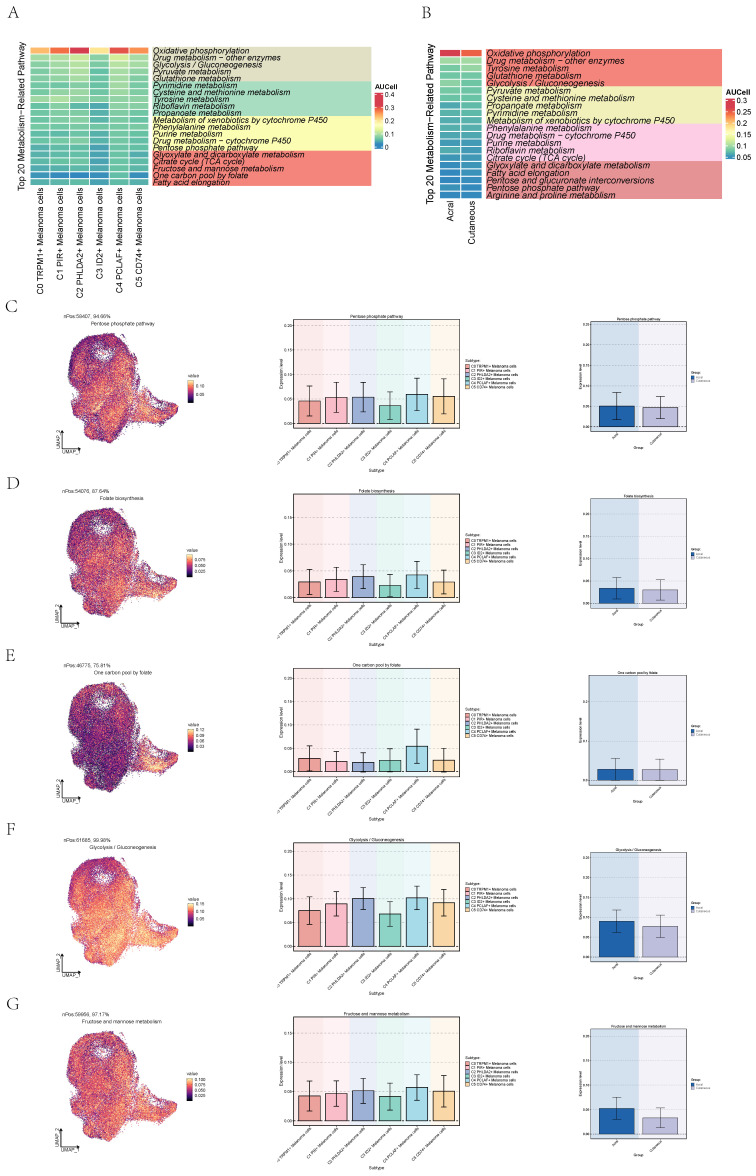
** Activity of metabolism-related pathways in melanoma cells. (A)** Heatmap showed activity of the top 20 metabolism-related pathways in C4 PCLAF+ Melanoma cells.** (B)** Heatmap showed activity of the top 20 metabolism-related pathways in acral melanoma cells.** (C)** UMAP diagram(left) and bar graphs (middle and right) showed activity of Pentose phosphate pathway in melanoma cells.** (D)** UMAP diagram(left) and bar graphs (middle and right) showed activity of Folate biosynthesis in melanoma cells.** (E)** UMAP diagram(left) and bar graphs (middle and right) showed activity of One carbon pool by folate in melanoma cells.** (F)** UMAP diagram(left) and bar graphs (middle and right) showed activity of Glycolysis / Gluconeogenesis in melanoma cells.** (G)** UMAP diagram(left) and bar graphs (middle and right) showed activity of Fructose and mannose metabolism in melanoma cells.

We quantified the activity of metabolic pathways to analyze the metabolic profiles in different subpopulations of melanoma cells. We showed the top 20 metabolic pathways ranked within the C4 subtype and AM using heatmaps (**Figure 7A,B**). Pathways pertinent to cellular energy provision and Cell proliferation are notably upregulated in melanoma cells, particularly within the C4 subpopulation^42^-^44^. Moreover, the metabolism of glutathione metabolism, a pathway associated with cellular antioxidation, is also highly expressed in melanoma cells, particularly within the C2 and C4 subpopulations^45^. Additionally, we observed that pyruvate metabolism, glycolysis/gluconeogenesis and oxidative phosphorylation were concurrently active in both C4 PCLAF+ Melanoma cells and AM. These pathways can provide energy for the proliferative activities of active cells and furnish raw materials. Subsequently, we proceeded to further analyze the significantly upregulated metabolic pathways in C4 subtype and AM.

Pentose phosphate pathway exhibited high expression in both C4 subtype and AM (**Figure 7C**), which was associated with cellular proliferation, not only does it provide energy for cellular proliferation, but its intermediates also serve as essential raw materials. The elevated expression of this metabolic pathway in C4 implied its proliferative function within the AM subgroup. Folate biosynthesis and one carbon pool by folate were associated with nucleic acid synthesis and were active in both the C4 subgroup and AM cells (**Figure 7D, E**). Glycolysis / Gluconeogenesis and fructose and mannose metabolism can provide energy for active cancer cells (**Figure 7F,G**). All of the aforementioned were correlated with the active proliferative state of cells.

### CellChat analysis between cells

**Figure 8:**
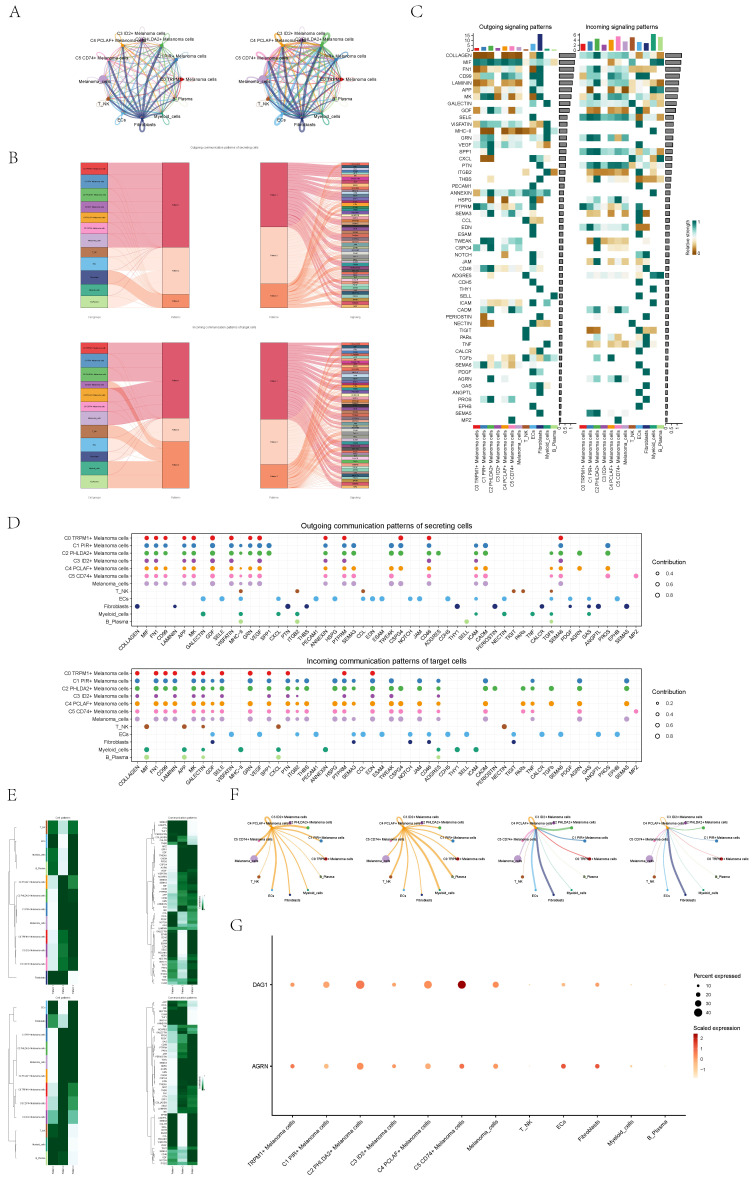
** CellChat analysis among all cells. (A)** Circle plots showed the number (left) and strength (right) of interactions between all cells.** (B)** Sankey diagrams showed inferred outgoing communication patterns of secreting cells, showed correspondence between inferred potential patterns, cell populations, and signaling pathways. Left: incoming Sankey diagram, right: outgoing Sankey diagram.** (C)** Heatmap showed incoming and outgoing signaling intensities for the all cellular interactions.** (D)** Outgoing contribution bubble plot and incoming contribution bubble plot showedcellular communication patterns among various cell subpopulations of melanoma cells and other cells. **(E)** Heatmap showed pattern recognition of incoming cells (top), and outgoing cells (bottom) among all cells. **(F)** Circle plots showed the number (left) and strength (right) of interactions between C4 melanoma cells and other cells. Top:C4 melanoma cells served as source cell, bottom:C4 melanoma cells served as target cell.

To systematically elucidate complex cellular responses, we probed cell-to-cell relationships and ligand-receptor communication networks to better understand cell interactions. Using CellChat analysis, we initially established intercellular communication networks among various cell types, such as Myeloid cells, Fibroblasts, T_NK cells, and different subgroups of melanoma cells. After that, we calculated the number of interactions, represented by the thickness of the "line" connecting two cell types, indicating a higher number of interaction pathways with thicker lines, and the strength of interactions, depicted by the weight of the "line", where thicker lines represent stronger interaction strength (**Figure 8A**).

We utilized gene expression pattern analysis methods provided by CellChat to investigate the interaction between cells and signaling pathways. First, we determined the correspondence between inferred potential communication patterns and groups of cells to decipher the communication patterns. This analysis revealed three outgoing communication patterns of secreting cells and three incoming communication patterns of target cells: pattern 1 of outgoing communication (melanoma cells), pattern 2 of outgoing communication (T_NK cells, B_Plasma cells, Myeloid cells, ECs),pattern 3 of outgoing communication (Fibroblasts), pattern 1 of incoming communication (the majority of melanoma cells.), pattern 2 of incoming communication(ECs and Fibroblasts), and pattern 3 of incoming communication(C3 melanoma cells, T_NK cells, B_Plasma cells, Myeloid cells). Moreover, we used CellChat to detect essential signals across various cell group based on non-negative matrix decomposition. For example, the predominant signaling in outgoing melanoma cells was typified by pattern 1, encompassing various pathways such as AGRN, VEGF, CD99, and others. Output T_NK cells, B_Plasma cells, Myeloid cells, ECs were characterized by pattern 2, which represented pathways such as SELE, CCL, EDN and CDH5. Conversely, communication patterns of target cells indicated that incoming melanoma cell signaling was predominantly characterized by pattern 1, which including pathways such as CD99 and TNF as well as PARS, TGFb, AGRN, and MPZ (**Figure 8B**). Subsequently, we juxtaposed the potency of signaling molecules across subpopulations, revealing a heightened intensity of CD99 and MK within the C4 PCLAF+ Melanoma cells subtype (**Figure 8C**).

We observed that AGRN is primarily secreted by C2 PHLDA2+ Melanoma cells subgroup and C4 PCLAF+ Melanoma cells subtype, with its main target cells (receptors) were the majority of melanoma cells. Besides, TNFb is predominantly secreted by various immune cells, ECs, Fibroblasts and a subset of melanoma cells, and its target cells were C2 PHLDA2+ Melanoma cells and C4 PCLAF+ Melanoma cells. These conclusions could also be corroborated in bubble plots (**Figure 8D**) and heatmaps (**Figure 8E**).

Lastly, we individually analyzed the interaction between C4 PCLAF+ Melanoma cells subtype and other cell types (**Figure 8F**). It showed that C4 cells exhibited intricate communication with both melanoma cells and other cells.

### Analysis of ARGN and TGFb signal pathway

**Figure 9:**
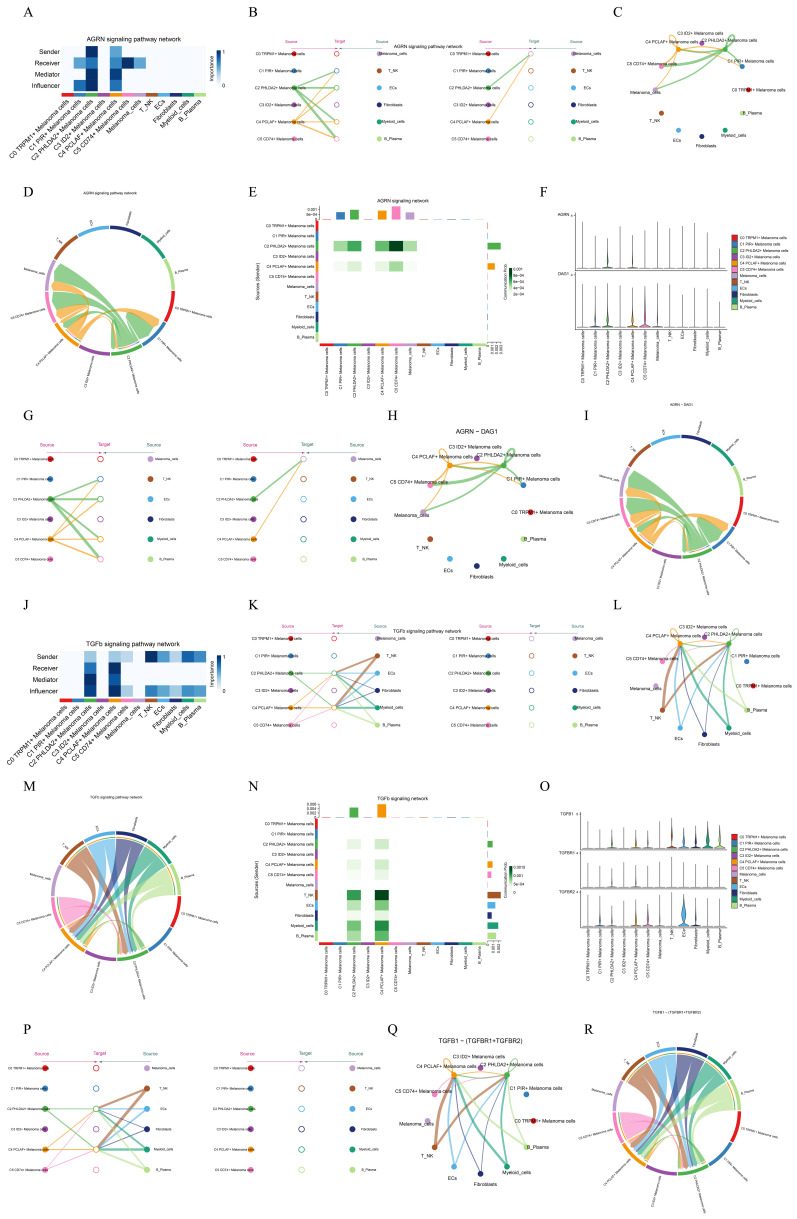
** Visual analysis of two signaling pathways. (A)** Heatmap demonstrated the centrality score of the AGRN signaling pathway network, showing the relative importance of each cell group.** (B)** Hierarchical diagram showed the interactions between melanoma cells and other cells in the AGRN signaling pathway. Solid and hollow circles indicated source and target cell types, respectively. The edge color of the middle circle was consistent with the signal source. **(C)** Circle plots showed the interactions between C4 melanoma cells and other cells in the AGRN signaling pathway when C4 melanoma cells were selected as the RECEIVER. **(D)** Circle plot showed the cellular interactions of the AGRN signaling pathway when C4 melanoma cells were selected as the RECEIVER. **(E)** Heatmap showed the cell interactions of the ARGN signaling pathway. **(F)** Violin plot showed the cellular interactions of the ARGN-DGA1 signaling pathway. **(G)** Hierarchical diagram showed the interactions between melanoma cells and other cells in the AGRN-DAG1 signaling pathway. Solid and hollow circles indicated source and target cell types, respectively. The edge color of the middle circle was consistent with the signal source. **(H)** Circle plots showed the interactions between C4 melanoma cells and other cells in the AGRN-DAG1 signaling pathway when C4 melanoma cells were selected as the RECEIVER. **(I)** Circle plot showed the cellular interactions of the AGRN-DAG1 signaling pathway when C4 melanoma cells were selected as the RECEIVER. **(J)** Heatmap demonstrated the centrality score of the TGFb signaling pathway network, showing the relative importance of each cell group. **(K)** Hierarchical diagram showed the interactions between melanoma cells and other cells in the TGFb signaling pathway. Solid and hollow circles indicated source and target cell types, respectively. The edge color of the middle circle was consistent with the signal source. **(L)** Circle plots showed the interactions between C4 melanoma cells and other cells in the TGFb signaling pathway when C4 melanoma cells were selected as the RECEIVER.** (M)** Circle plot showed the cellular interactions of the TGFb signaling pathway when C4 melanoma cells were selected as the RECEIVER. **(N)** Heatmap showed the cell interactions of the TGFb signaling pathway. **(O)** Violin plot showed the cellular interactions of the TGFb-(TGFBR1+TGFBR2) signaling pathway. **(P)** Hierarchical diagram showed the interactions between melanoma cells and other cells in the TGFb-(TGFBR1+TGFBR2) signaling pathway. Solid and hollow circles indicated source and target cell types, respectively. The edge color of the middle circle was consistent with the signal source.** (Q)** Circle plots showed the interactions between C4 melanoma cells and other cells in the TGFb-(TGFBR1+TGFBR2) signaling pathway when C4 melanoma cells were selected as the RECEIVER.** (R)** Circle plot showed the cellular interactions of the TGFb-(TGFBR1+TGFBR2) signaling pathway when C4 melanoma cells were selected as the RECEIVER.

In order to explore the function way of ARGN signal pathway, the ARGN signal pathway was visually analyzed. We identified the cell types as the sender, receiver, medium and influencer of ARGN signaling-mediated intercellular communication according to the relative importance of each cell type based on the algorithm, which was called "centrality measurement". As could be seen from the figure, C2 PHLDA2+ Melanoma cells and C4 PCLAF+ Melanoma cells had the highest expression on the ARGN signaling pathway (**Figure 9A**). If we set all 10 identified cell types in melanoma as the source cells of ARGN, and set the five cell types listed on the left in **Figure 9B** as potential target cells, then the hierarchical diagram showed that ARGN released by C2 PHLDA2+ Melanoma cells and C4 PCLAF+ Melanoma cells, and both C2 and C4 cells could target melanoma cells expect C3 ID2+ Melanoma cells (**Figure 9B**). The ligand-receptor between tumor epithelial cells and other cells displayed by chord diagram and network digram could also serve to corroborate this assertion (**Figure 9C,D**). These results indicated that the majority of melanoma cells in melanoma may be the target of AGRN, but C2 PHLDA2+ Melanoma cells and C4 PCLAF+ Melanoma cells subgroup had different targeting intensities (line width between cells) for AGRN. The specific situation of cell-to-cell interaction in AGRN signaling pathway was shown in the figure (**Figure 9E**). The role of AGRN-DAG ligand-receptor molecules in cell interactions was showed in diagrams(**Figure 9F-I**).It was same to the function way of AGRN signal pathway. This suggested that AGRN primarily acted by targeting DAG in melanoma cells.

We employed the same methodology to analyze the TGFb signaling pathway. We found that TGFb can be secreted by immune cells (T_NK cells, B_Plasma cells, Myeloid cells), ECs, Fibroblasts, C2 PHLDA2+ Melanoma cells, C4 PCLAF+ Melanoma cells and C5 CD74+ Melanoma cells, and targeting C2 PHLDA2+ Melanoma cells and C4 PCLAF+ Melanoma cells (**Figure 9J-M**). The specific cell-to-cell interaction in the TGFb signaling pathway was depicted in the figure (**Figure 9N**). TGFb primarily achieved cell-to-cell communication by targeting TGFBR1 and TGFBR2 (**Figure 9O-R**).

## Discussion

In this study, we employed single-cell RNA sequencing (scRNA-seq) to comprehensively delineate the cellular heterogeneity of human cervical cancer (CC). Utilizing scRNA-seq technology, we identified all cell types present in CC, including melanoma cells, T_NK cells, fibroblasts, endothelial cells (ECs), myeloid cells, and B plasma cells. Among these, melanoma cells emerged as the most abundant cell type. Given previous research demonstrating acral melanoma's highly malignant nature with characteristics of early metastasis and rapid dissemination, our focus shifted to studying acral melanoma cells^2^. We characterized melanoma cells and conducted dimensionality reduction clustering, revealing six distinct cell subgroups: C0 TRPM1+ Melanoma cells, C1 PIR+ Melanoma cells, C2 PHLDA2+ Melanoma cells, C3 ID2+ Melanoma cells, C4 PCLAF+ Melanoma cells, and C5 CD74+ Melanoma cells. We noted that the proportion of cells in the AM far exceeded that of CM in the C4 subgroup.

Subsequently, we analyzed the enrichment of gene and cell stemness in different melanoma cell subpopulations. The results showed that C4 PCLAF+ Melanoma cells had high cell stemness and strong proliferation ability. The utilization of Slingshot and Monocle allowed for the demonstration of the differentiation trajectory of melanoma cells along a pseudo-temporal sequence. C3 ID2+ Melanoma cells were positioned at the initiation stage of the differentiation trajectory while C4 PCLAF+ Melanoma cells resided at the terminal end of the differentiation trajectory.

Regarding the nomenclature of C4 PCLAF+ Melanoma subtype, previous research suggests that PCLAF, also recognized as PAF15/KIAA0101, is a nucleoprotein with a molecular weight of 15 kDa. It was initially identified through yeast two-hybrid screening and has the ability to bind to PCNA (proliferating cell nuclear antigen)^46^. It is widely overexpressed in most human cancers and serves as a predominant regulator of tumor progression^47^. PLP2 has been implicated in accelerating the G1/S transition of cell cycle by activating the E2F1/PTTG1 signaling pathway, thus facilitating cell cycle progression^48^. Therefore, we hypothesized that the C4 PCLAF+ Melanoma cells subgroup is intricately linked to the progression of the tumor.

To analyze the characteristics of the C4 subgroup, we compared the expression level of transcription factor and activity of metabolic pathway across different subgroups. Compared to other subgroups, the C4 subgroup exhibited high expression of transcription factors associated with cell proliferation, such as E2F2, E2F7, and E2F8^37^-^41^. Metabolic pathways associated with cell proliferation and cellular energy production, including pentose phosphate pathway and glycolysis / gluconeogenesis, were more active in the C4 subgroup, which also suggests its higher malignancy level.

To explore interactions between the C4 PCLAF+ Melanoma cell subgroup and other cell types, CellChat communication pattern analysis was employed to unveil coordinated responses among different cell types. Distinct cell types can simultaneously activate either common, cell type-independent signaling transduction pathways or distinct, cell type-specific signaling transduction pathways. This methodology is utilized for inferring, analyzing, and visualizing intercellular communication from given scRNA-seq data^49^. Through the application of CellChat to depict the relationships between the melanoma cell subgroup and other cell types, three patterns were identified along with their corresponding signal pathway expressions. Notably, the AGRN signaling pathway corresponds to Melanoma cells pattern 1 and targets almost all melanoma cells, signifying its crucial role as a significant signaling pathway. The presentation of the subgroups on the AGRN signaling pathway reveals that the C4 PCLAF+ Melanoma cells and C2 PHLDA2+ Melanoma cells subgroup has the highest quantity and centralization score, providing evidence of a robust association between this pathway and the C4 PCLAF+ Melanoma cells subgroup. This confirmation underscores the significance of the C4 PCLAF+ Melanoma cells as the focal point in this study. Additionally, we found that the TGFb signaling pathway only targets the C4 PCLAF+ Melanoma cells and C2 PHLDA2+ Melanoma cells subgroup, which suggests that this pathway plays an important role in the C4 subgroup.

## Conclusion

In summary, our study identified crucial subtypes C3 ID2+ melanoma cells and C4 PCLAF+ Melanoma cells in acral melanoma. Furthermore, we analyzed the characteristics of the subtypes as well as the interactions with other cells. Our research unveils potential therapeutic targets for melanoma, providing valuable resources and deeper insights into the occurrence and development of melanoma.

## Supplementary Material

Supplementary figure.
