# Supplementary figures and images for: Single-cell analysis unveils cell subtypes of acral melanoma cells at the early and late differentiation stages
Source: J Cancer. 2025 Jan 1;16(3):898–916. doi: 10.7150/jca.102045 (PMC11705046; doi:10.7150/jca.102045)

A B

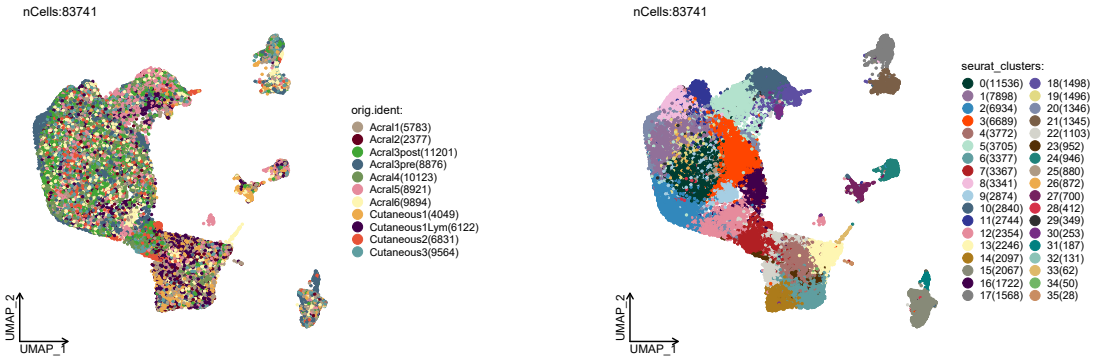

C

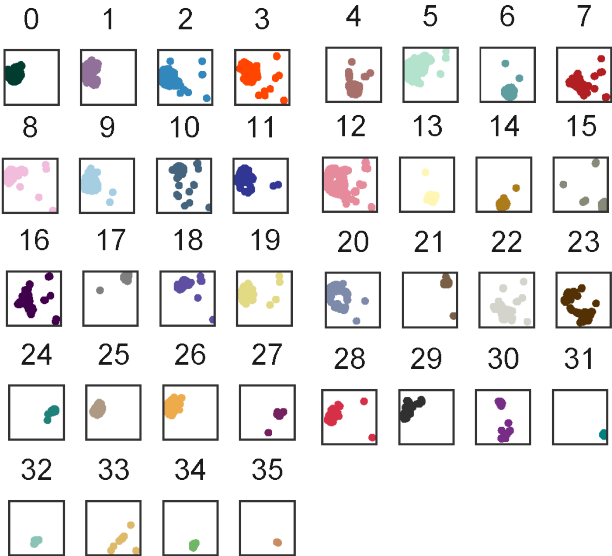

D

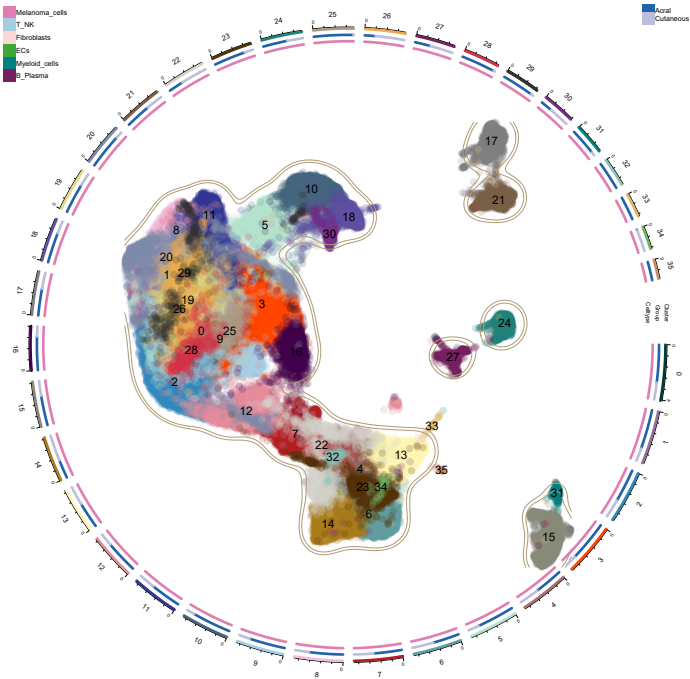

Supplement: Supplementary file 1 — Supplementary figure. [file jcav16p0898s1.pdf]
